# Cooperative Intelligent Transport Systems: The Impact of C-V2X Communication Technologies on Road Safety and Traffic Efficiency

**DOI:** 10.3390/s25072132

**Published:** 2025-03-27

**Authors:** Jingwen Wang, Ivan Topilin, Anastasia Feofilova, Mengru Shao, Yadong Wang

**Affiliations:** 1 Don School, International Education College, Shandong Jiaotong University, Jinan 250357, China; 213023@sdjtu.edu.cn (J.W.); 203075@sdjtu.edu.cn (Y.W.); 2 Faculty of Road and Transportation, Don State Technical University, 1 Gagarin sq., Rostov-on-Don 344000, Russia; ivan_top@mail.ru (I.T.); feofilowa@mail.ru (A.F.); 3 Urban and Data Science, Graduate School of Advanced Science and Engineering, Hiroshima University, Higashi-Hiroshima 739-8511, Japan

**Keywords:** ITS, MATLAB, C-V2X, following model, signal attenuation, autonomous vehicle, traffic simulation, road safety, SUMO

## Abstract

The advancement of intelligent road transport represents a promising direction in the evolution of transportation systems, aimed at improving road safety and reducing traffic accidents. The integration of artificial intelligence, sensors, and machine vision systems enables autonomous vehicles (AVs) to rapidly adapt to changes in the road environment, minimizing human error and significantly reducing collision risks. These technologies provide continuous and highly precise control, including adaptive acceleration, braking, and maneuvering, thereby enhancing overall road safety. Connected vehicles utilizing C-V2X (Cellular Vehicle-to-Everything) communication primarily feature real-time operation, safety, and stability. However, communication flaws, such as signal fading, time delays, packet loss, and malicious network attacks, can affect vehicle-to-vehicle interactions in cooperative intelligent transport systems (C-ITSs). This study explores how C-V2X technology, compared to traditional DSRC, improves communication latency and enhances vehicle communication efficiency. Using SUMO simulations, various traffic scenarios were modeled with different autonomous vehicle penetration rates and communication technologies, focusing on traffic conflict rates, travel time, and communication performance. The results demonstrated that C-V2X reduced latency by over 99% compared to DSRC, facilitating faster communication between vehicles and contributing to a 38% reduction in traffic conflicts at 60% AV penetration. Traffic flow and safety improved with increased AV penetration, particularly in congested conditions. While C-V2X offers substantial benefits, challenges such as data packet loss, communication delays, and security vulnerabilities must be addressed to fully realize its potential. Future advancements in 5G and subsequent wireless communication technologies are expected to further reduce latency and enhance the effectiveness of C-ITSs. This study underscores the potential of C-V2X to enhance collision avoidance, alleviate congestion, and improve traffic management, while also contributing to the development of more reliable and efficient transportation systems. The continued refinement of simulation models and collaboration among stakeholders will be crucial to addressing the challenges in CAV integration and realizing the full benefits of connected transportation systems in smart cities.

## 1. Introduction

Autonomous transport is innovative technology with significant potential to enhance road safety and reduce the frequency of road traffic accidents. The integration of such vehicles into the overall transport system, combining the use of artificial intelligence, machine vision, and various sensors, opens new horizons in traffic flow management. The application of these technologies minimizes the impact of human factors, which are one of the main causes of accidents, leading to a substantial improvement in road safety [1].

Research analysis indicates that an increase in the proportion of AVs on roads significantly reduces the number of conflict situations. For example, with the penetration rate of connected and autonomous vehicles (CAVs) ranging from 18.9% to 94.1%, a progressive decrease in the number of road conflicts is observed [2]. Additionally, the introduction of AVs into the traffic flow contributes to a reduction in vehicle delays of 26% to 74.2%, depending on their presence in the overall traffic volume within the transport network [3].

The role of Cellular Vehicle-to-Everything (C-V2X) technology in improving road safety and traffic efficiency is a pressing issue in the present day. A well-known framework for analyzing C-V2X robustness under imperfect channel state information demonstrates how adaptive power schemes and metrics such as hazard indicators can optimize communication reliability in dynamic environments, contributing to safer and more efficient transportation systems [4]. In addition, advanced data processing frameworks adapted for 5G C-V2X networks have significantly increased data processing speed and reduced CPU load, meeting the real-time needs of cooperative intelligent transportation systems [5].

The integration of C-V2X with other communication protocols such as LoRa and DSRC has shown promising results in hybrid systems that leverage the strengths of each technology, such as scalability and obstacle avoidance, to maximize the safety and efficiency of urban intersections [6]. This adaptability is further supported by the optimization of scenario-dependent parameters, as investigated in a study using Simulation of Urban MObility (SUMO) [7] and OMNET++ [8] to simulate dense traffic conditions, where C-V2X effectively addressed latency and scalability issues [9]. The widespread adoption of C-V2X worldwide has also been noted as a driving force for autonomous driving and predictive collision avoidance, enabling real-time data exchange and improved traffic management [8]. Security remains a critical issue, as research highlights the need for robust protocols to protect data integrity and user privacy in C-V2X networks, ensuring trust and functionality in real-world deployments [10]. Innovations in C-V2X infrastructure services, such as optimal activation zones, have demonstrated improved communication efficiency and scalability, paving the way for a variety of transportation applications in dense traffic environments [11]. Practical deployments, such as Signal Phase and Timing (SPAT) applications, show how traffic signal data can guide AVs, improving route planning and safety [12].

C-V2X also plays an important role in protecting vulnerable road users (VRUs) through collision warning systems that use smartphone-enabled communications to alert pedestrians and drivers with exceptional accuracy and low latency [13]. Further research on resource allocation in C-V2X Mode 4 has focused on addressing communication inefficiencies in congested urban areas, demonstrating improved message reliability and reduced collision probability [14]. This focus on latency extends to vehicle-to-pedestrian (V2P) communications, where Multi-access Edge Computing (MEC) servers significantly reduce latency, improving safety at urban intersections [15,16].

Scalability studies highlight the ability of C-V2X to cope with high vehicle densities in urban environments, reinforcing its role as a cornerstone for intelligent transportation systems. Optimized resource management in high-congestion scenarios enables efficient communications even in challenging environments [17]. Finally, advanced network configurations and hybrid approaches highlight the adaptability of C-V2X and its vital contribution to scalable, secure, and reliable ITS applications in modern urban mobility [18,19].

The simulation of autonomous vehicle movement is an essential tool for analyzing and predicting its impact on the safety and efficiency of the transport system. Such experiments allow for the consideration of various traffic scenarios, including mixed flows consisting of both traditional vehicles and AVs, as well as the analysis of potential risks and the development of measures to mitigate them. For instance, the use of models based on molecular dynamics significantly improves safety characteristics in vehicle-following, ensuring more stable and safe behavior on the road [20]. One of the key aspects of enhancing safety is also the development of innovative traffic management strategies, such as game theory-based algorithms that optimize lane-changing processes, minimizing collision risks [21].

Furthermore, the use of data from connected vehicles enables real-time information exchange between drivers and other road users, which also contributes to improved road safety [22]. For example, in China, C-V2X (Cellular Vehicle-to-Everything) technology is currently being actively researched. This system of wireless communication for vehicles is based on the evolution of cellular technologies such as 4G and 5G. This platform can be used to develop ITS services that provide comprehensive communication and efficient information exchange between vehicles, road infrastructure, base stations, and cloud platforms [23]. It is expected that this technology will eliminate up to 80% of current road traffic accidents due to more accurate and timely data exchange between all elements of the transport system [24].

To evaluate the effectiveness of cellular communication technologies in ensuring road safety, parameters such as the data packet loss rate and end-to-end delay are used.

The data packet loss rate reflects the proportion of packets sent from the source that do not reach the recipient. This parameter is important for assessing communication reliability and service quality.

End-to-end delay represents the time required for a packet to travel from the source to the destination. This is a critical parameter, especially for applications requiring rapid response, such as safety and automated driving systems.

**Figure 1:**
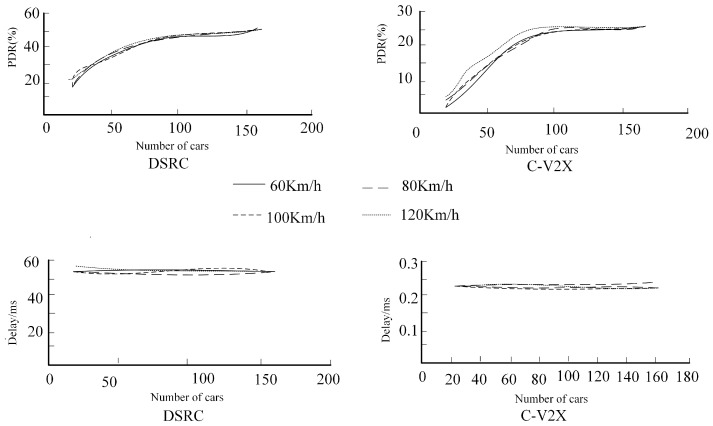
Comparison of data packet loss rate and end-to-end delay simulation for C-V2X and DSRC technologies.

Experiments conducted on the OMNET++ simulation platform, combining the Veins (Vehicles in Network Simulation) algorithm for modeling connected vehicles and SUMO (Simulation of Urban Mobility) for traffic simulation, showed that the data packet loss rate increased with the number of vehicles and their speed. The packet loss rate in DSRC (Dedicated Short-Range Communications) technology was higher than that in C-V2X, as shown in Figure 1. At the same time, the data transmission delay in C-V2X was significantly lower than that in DSRC, amounting to 0.25 ms and 50–55 ms, respectively [25]. So, it is found that a large amount of research is focused on the safety, cost, efficiency, and infrastructure aspects of autonomous driving vehicles. A few studies are based on the potential impact and outcomes of autonomous driving vehicles on urban land use and urban form. There is little research on whether autonomous driving vehicles can meet the transportation needs of different cities.

Thus, C-V2X communication technology should become the international standard for communication on highways to enhance the safety and efficiency of ITSs.

The essence of vehicle-to-vehicle communication technology offers numerous advantages for the management of connected vehicles. However, it is important to acknowledge that certain uncertainties, such as packet loss or temporal delays, as well as the characteristics of the communication channel, including bandwidth limitations inherent in the existing Dedicated Short-Range Communications (DSRC) technology, may compromise the accuracy of managing C-ITSs. These factors can also impact driving safety. Consequently, it is imperative to investigate the effects of communication delays, packet loss, and other related factors on the behavior of intelligent vehicles [26,27,28,29].

The Acceleration-Impairment Driver Model (AIDM) significantly advances traditional car-following models like the Intelligent Driver Model (IDM) and Gipps Model by addressing critical gaps in communication-aware vehicle dynamics. Unlike conventional approaches, AIDM explicitly integrates real-world communication impairments—such as packet loss and delays—through parameters like the acceleration loss coefficient (γ) and stochastic error (ξ). These parameters quantify the degradation in communication quality, a necessity underscored by Shui et al. (2024) [4], who emphasize adaptive strategies for C-V2X under imperfect channel conditions. Traditional models like the IDM and Gipps, in contrast, assume flawless communication, ignoring practical challenges like the delays inherent in DSRC systems. Conversely, traditional models like Gipps’ over-conservative approach or the IDM’s reactive logic heighten collision risks under poor communication.

The AIDM further distinguishes itself through adaptability, enabling the calibration of γ to reflect specific communication protocols (see Table 1). This flexibility aligns with scalable C-V2X transactional services demonstrated by Zaman et al. (2022) [12], highlighting the AIDM’s capacity to adapt to diverse technologies. Older models, reliant on static parameters, lack this dynamic tuning, rendering them ineffective in heterogeneous communication environments (see Table 2).

To address the adequacy and accuracy of the AIDM compared to existing models, the following structured analysis is provided in Table 3.

The AIDM demonstrates high adequacy and accuracy in modeling V2X communication impacts on vehicle behavior, outperforming traditional models in safety and efficiency metrics. Its explicit incorporation of communication impairments (via *γ* and *ξ*) addresses a critical need in C-ITS research. 

Traditional traffic simulation models, such as the IDM, primarily focus on vehicle dynamics under ideal communication conditions. However, these models often overlook the impact of communication impairments—such as data packet loss and latency—that are prevalent in real-world ITS environments. To address this gap, we propose the use of the AIDM, an extension of the IDM, which incorporates communication-induced acceleration impairments. This model provides a more realistic representation of vehicle-following behavior under varying communication conditions.

Complementing the AIDM, the SUMO platform offers a comprehensive environment for modeling and simulating large-scale traffic networks. SUMO’s capabilities allow for the simulation of intermodal traffic systems, including road vehicles, public transport, and pedestrians, facilitating the analysis of complex traffic scenarios and the evaluation of ITS strategies.

By integrating the AIDM within the SUMO simulation environment, our study uniquely captures the effects of communication impairments on vehicle behavior and traffic flow. This integration enables the assessment of various communication technologies, such as C-V2X, under realistic traffic conditions.

## 2. Materials and Methods

**Figure 2:**
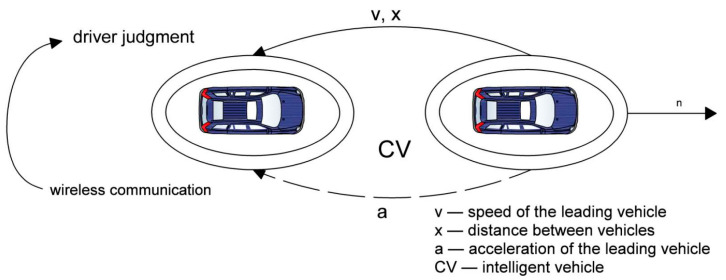
The configuration of a car-following model for a connected vehicle.

The initial stage of the investigation involved the search for and development of a car-following model for vehicles trailing a connected autonomous vehicle within a cooperative ITS. The general interaction scheme of connected vehicles for studying the car-following model is illustrated in Figure 2.

In order to analyze the effects of data packet loss and delay on the behavior of connected vehicles within a C-ITS, an AIDM was formulated. This model extends the capabilities of the IDM, incorporating the effects of deteriorated data transmission performance in real-world traffic scenarios and aligning more closely with vehicle behavior in V2X environments. The original IDM [32,33] is defined by Equation (1):

(1) $$a_{s}=a_{m}\left[1-{\left(\frac{v_{S}}{v_{0}}\right)}^{\delta }-{\left(\frac{s_{0}+v_{s}T+v_{s}{\Delta v\left(2\sqrt{a_{m}b}\right)}^{-1}}{\Delta x-l}\right)}^{2}\right]$$

where

$a_{s}$—the acceleration of the following vehicle;
$a_{m}$—the maximum acceleration of the vehicle;
$v_{0}$—the desired speed;
$v_{S}$—the speed of the following vehicle;
δ—the acceleration exponent of the vehicle;
$s_{0}$—the minimum (gap) spacing between vehicles, measured from the rear bumper of the leading vehicle to the front bumper of the following vehicle;
T—the safe time headway between vehicles, measured from front bumper to front bumper;
Δv—the speed difference between adjacent following vehicles;
Δx—the distance traveled;
b—the desired deceleration;
*l*—the length of the vehicle.

The consideration of data packet loss and time delay can be addressed using an Acceleration-Impairment Model for a connected vehicle. The structure of the Acceleration-Impairment Model for a vehicle connected to the C-ITS is defined as follows (2):

(2) $$a_{loss}\left(t\right)=10\cdot \gamma \cdot \mathrm{lg}\left(\frac{{\Delta x}_{n}\left(t\right)}{x_{0}}\right)+\xi$$

where

$a_{loss\;}\left(t\right)$—the magnitude of the signal attenuation of acceleration from a leading vehicle to a following vehicle at a distance of *s(t)* between the leading and following vehicles at the time *t*;
$x_{0}$—the relative distance to the anchor point, given by the minimum following distance;
γ—the exponential coefficient of acceleration impairment, which largely depends on travel duration, vehicle type, and spatial road conditions;
ξ—random error (in calculation).

With *x*_0_ = 1 m and neglecting the stochastic error, the resulting adapted AIDM takes the following form (3):

(3) $$a_{loss}\left(t\right)=\gamma \cdot \mathrm{lg}\left({\Delta x}_{n}\left(t\right)\right)+\xi$$

With the IDM car-following model refined using the AIDM, the resulting car-following model for an intelligent connected vehicle is given by (4):

(4) $$\left\{\begin{array}{c} a_{n}\left(t+T\right)=a_{IDM}+a_{n-1}\left(t\right)+a_{loss}\left(t\right)\\ v_{n}\left(t+T\right)=v_{n}\left(t\right)+a_{n}\left(t+T\right)\cdot T \end{array}\right.$$

where

$a_{IDM}$—the acceleration values determined by the *IDM*;
$a_{n-1}\left(t\right)$—the acceleration value of the leading vehicle at a given time, *t*;
T—the refresh interval;
$a_{n}\left(t+T\right)$—the refreshed acceleration values for connected vehicles in the ITS framework;
$v_{n}\left(t+T\right)$—updated speed values for vehicles connected to a communication network in the cooperative ITS.

Thus, the core idea is that the AIDM adjusts the acceleration of a connected vehicle based on the following:

Real-time communication quality: Modeled by *γ*, which scales the logarithmic term $\mathrm{lg}\left({\Delta x}_{n}\left(t\right)\right)$.
Leading vehicle dynamics: Incorporates the acceleration of the preceding vehicle, $a_{n–1}\left(t\right)$, to enable cooperative behavior.
Time delays and packet loss: Reflected in the refresh interval T and stochastic error ξ.

The process of the AIDM can be presented by the following steps:

**Initialization**:

- Set protocol-specific parameters (*γ*, *ξ*, packet loss probability, delay).
- Initialize vehicle states (position, speed, acceleration of ego and leading vehicles).

**Communication Process**:

- **Packet Loss**: Randomly discard data packets based on the loss probability (e.g., 5% for DSRC).
- Delay and Noise: Apply protocol-specific latency (e.g., 0.25 ms for C-V2X) and add random error (*ξ*) to received acceleration data.

**Acceleration Calculation**:

- Base IDM Acceleration: Compute the acceleration using the classic IDM formula, which depends on the speed, gap to the leading vehicle, and relative speed.
- Acceleration Impairment Term: Adjust the IDM output using: *a*_loss_ = *γ*⋅log(Δ*x*), where Δ*x* is the gap to the leading vehicle. This term amplifies or dampens acceleration based on communication quality.
- Total Acceleration: Combine the IDM output, impairment term, and stochastic error: total = a_IDM_ + a_loss_ + *ξ*.

**Vehicle State Update**:

- Enforce physical limits on the acceleration (*b*_max_ ≤ *a*_total_ ≤ *a*_max_) and speed (0 ≤ *v* ≤ *v*_max_).
- Update the ego vehicle’s position and speed using kinematic equations.

**Iteration**:

- Repeat the process for each time step, dynamically adjusting to changing communication conditions and leading vehicle behavior.

Following the empirical calibration of *γ*, the exponential coefficient of acceleration loss, it was found [34] that its value is 1.9 for vehicles employing C-V2X technology, while vehicles with DSRC and related technologies experience substantially higher data packet loss and delay.

Therefore, the current focus of the authors is on exploring the impact of communication technology on traffic safety through the manipulation of the exponential coefficient of acceleration loss, *γ*.

Another part of this article focuses on evaluating the impact of C-V2X technology on traffic safety through simulations in the SUMO software environment due to its strengths in modeling CAVs and its ability to simulate mixed traffic environments. SUMO is an open-source platform that supports the simulation of both human-driven vehicles and AVs, which is essential for understanding their interaction in real-world traffic scenarios.

A key advantage of SUMO is its integration with communication simulators like OMNeT++ and ns-3, enabling us to model Vehicle-to-Everything (V2X) communication systems and assess the impact of communication impairments like latency and packet loss on CAV performance. This integration is crucial for our research, as communication quality is a central factor in traffic safety and efficiency.

SUMO’s capabilities are well-supported in the literature. According to [35], SUMO is widely used for CAV modeling due to its flexibility and scalability. Similarly, ref. [36] highlights its adaptability for representing complex traffic scenarios and integrating communication models, making it a top choice for simulating CAVs.

Accordingly, this study considers two traffic simulation scenarios.

### 2.1. A Traffic Composition That Consists Entirely of CAVs

**Figure 3:**
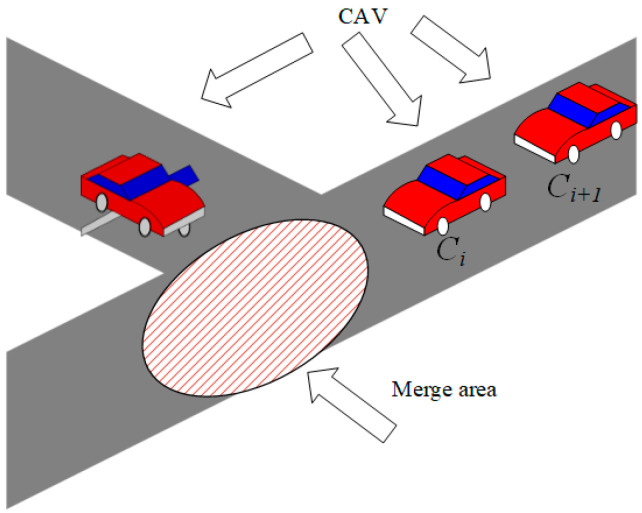
A cooperative merge scenario at the junction between a main highway and an on-ramp.

Under this condition, an intelligent collision avoidance model for AVs is described as follows:

At any point in time, *t*, given that $C_{i}$ is the lead vehicle, the time at which it is expected to arrive at the merge area, * $t_{merge,i}$, can be precisely calculated. The value for $t_{merge,i}$ can also be precisely calculated for the next CAV, $C_{i+1}$, upon its arrival at the merge area (see Figure 3).

When *C_i_* enters the merge zone, the goal to promote safety and effectiveness requires the speeds of the lead and lag CAVs to be equalized as much as possible using vehicle-to-vehicle communication to maintain a safe headway, i.e.,

(5) $$\left\{\begin{array}{l} min\left(v_{i,t_{\mathrm{merge},i}}-v_{i+1,t_{\mathrm{merge},i}}\right)\\ x_{i+1,t_{\mathrm{merge},i}}=x_{i,t_{\mathrm{merge},i}}-v_{i+1,t_{merge,i}}\cdot T_{\min,1} \end{array}\right.$$

where $v_{i},\iota_{merge,i}$ denotes the velocity of vehicle *i* at the time $t_{\;merge,\;}i$; $x_{i},\iota_{\;merge}$ represents the position of the vehicle at the time $t_{merge,}i$; and $T_{min,1}$ is the minimum allowed speed at the merge point for a main-road CAV and a ramp CAV.

If $t_{merge,i+1}-t_{merge,i}\leq T_{min,1}$, the time difference between the preceding and following vehicles passing through the merge point is less than the minimum allowable time headway for merging the main-road and ramp vehicles. Then, to avoid a collision between $C_{i+1}$ and $C_{i}$, $C_{i+1}$ has two options: (1) decelerate and establish a sufficient safety distance; (2) accelerate and pass through the merge point earlier. However, in practice, although $C_{i+1}\;$ can transmit acceleration information to the preceding vehicle and accelerate in conjunction with it, in a mixed traffic situation, the preceding vehicle is not necessarily a CAV and may not accept the information transmitted by $C_{i+1}$, making it difficult to ensure sufficient distance for acceleration, and therefore only option 1 is considered further.

To satisfy option 1, the acceleration of $C_{i+1}$ is calculated as follows (6):

(6) $$a_{i+1}=min\left\{min\left(a_{merge},a_{comfort}\right),a_{norm}\right\}$$

$a_{norm}$ is the normal acceleration, determined by driving regulations.
$a_{comfort}$ is the comfort acceleration.
$a_{merge}$ is the acceleration required for merging, determined from the equation of motion, i.e., (7):

(7) $$a_{merge}=\frac{x_{i,t_{merge,i}}-x_{i+1,t_{merge,i}}-v_{i+1,t_{merge,i}}\cdot T_{min,1}}{0.5\cdot {\left(t_{merge,i}-t\right)}^{2}+T_{min,1}\cdot \left(t_{merge,i}-t\right)}$$

### 2.2. Traffic Flow Comprises CAVs and Human-Driven Vehicles (HDVs)

**Figure 4:**
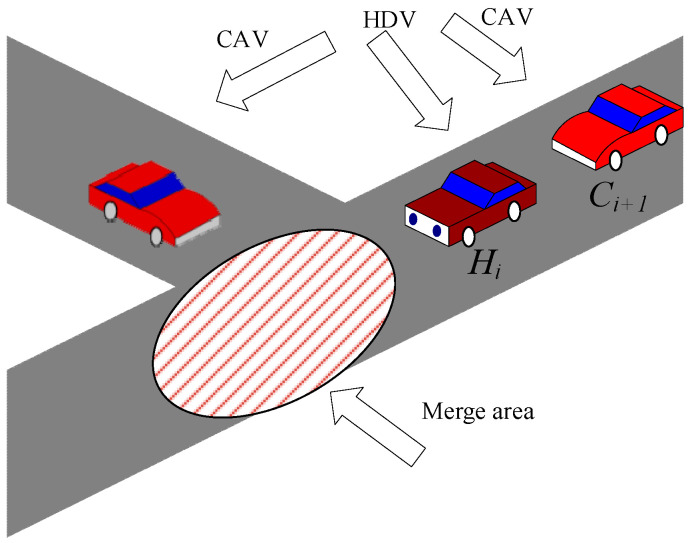
A mixed-traffic merge scenario at the junction between a main highway and an on-ramp.

In this context, the intelligent collision avoidance model is defined as follows:

When the preceding vehicle is $H_{i}$, the arrival time $H_{i}$ is estimated using a roadside unit or surrounding CAVs (see Figure 4), i.e.,

At the time $t_{merge,}$ the position of $C_{i+1}$ can be determined from its detected position and velocity at the detection time $t_{0}$ as follows (8):

(8) $$x_{i+1,t_{mep}}=x_{i+1,t_{0}}+v_{i+1,t_{0}}\cdot \left(t_{merge}-t_{0}\right)$$

The merging safety area for a ramp vehicle with respect to the main-road vehicle $C_{i+1}$ at the time $t_{merge}$ is defined by the velocity of $C_{i+1}$, i.e.,

(9) $$\left\{\begin{array}{l} x_{up}=x_{merge}-v_{i+1,t_{0}}\cdot T_{min,2}\\ x_{down}=x_{merge}+v_{i+1,t_{0}}\cdot T_{min,2} \end{array}\right.$$

where $x_{up}$ and $x_{down}$ are the upstream and downstream boundaries of the merging safety zone for the vehicle $C_{i+1}$, respectively; $x_{merge}$ is the upstream boundary of the merging zone; and $T_{min,2}$ is the minimum time headway for merging CAVs and HDVs. When $x_{i+1,l_{merge}}$ falls within the interval $\left[x_{up},x_{down}\right]$, to avoid a collision between $C_{i+1}$ and the preceding vehicle, $C_{i+1}$ also faces a similar issue of option selection as in scenario 1; therefore, only option 1 is considered further.

At the same time, to promote safety and efficient merging, it is deemed that the velocities of the main-road and ramp traffic should be harmonized to the maximum possible extent during the merging process, i.e.,

(10) $$min\left(v_{i,t_{\mathrm{merge}}}-v_{i+1,t_{\mathrm{merge}}}\right)$$

When $x_{i+1,l_{merge}}$ falls within the interval $\left[x_{up},x_{down}\right]$, in order to implement option 1, the required acceleration for $C_{i+1}$ is

(11) $$a_{i+1}=min\left\{min\left(a_{merge},a_{comfort}\right),a_{norm}\right\}$$

$a_{norm}$ is the normal acceleration, determined by driving regulations.
$a_{comfort}$ is the comfort acceleration.
$a_{merge}$ is the acceleration required for merging, determined from the equation of motion, i.e., (12):

(12) $$a_{merge}=\frac{x_{i,t_{merge}}-x_{i+1,t_{merge}}-v_{i+1,t_{merge}}\cdot T_{min,2}}{0.5\cdot {\left(t_{merge}-t_{0}\right)}^{2}+T_{min,2}\cdot \left(t_{merge}-t_{0}\right)}$$

Due to SUMO being an open-source package for continuous microscopic and multi-modal traffic modeling, the implementation of collaborative collision avoidance strategies for AVs is required, especially on highway ramps and in merger zones.

To explore a prospective transportation landscape dominated by AVs, the default parameters of the SUMO traffic simulator were recalibrated (see Table 4). This investigation utilized the Krauss car-following model, a standard component within SUMO. The parameter adjustments primarily targeted longitudinal vehicular dynamics, specifically focusing on the acceleration, deceleration, and gap acceptance behaviors. These behavioral aspects were operationalized and subsequently calibrated as pivotal parameters within the framework of the Krauss car-following model. The overarching aim of this parametric modification was to enable simulated vehicles to achieve maximum safe speeds while simultaneously maintaining strict adherence to safety regulations. This was realized through the implementation of controlled braking actions, constrained by predefined acceleration limits, which were triggered based on the relative positions and velocities of the preceding and following vehicles. A subsequent analysis will detail the specific customizable parameters of the Krauss car-following model that were subjected to manipulation in this study:

- Mingap: the offset to the leading vehicle when standing in a jam (in m).
- Accel: the acceleration ability of vehicles of this type (in m/s^2^).
- Decel: the deceleration ability of vehicles of this type (in m/s^2^).
- Emergency Decel: the maximum deceleration ability of vehicles of this type in case of an emergency (in m/s^2^).
- Sigma: the driver’s imperfection (between 0 and 1).
- Tau: the driver’s desired (minimum) time headway (reaction time) (in s) [37].

## 3. Results

The primary focus of this work was to analyze the impact of communication technologies on vehicular safety, which was accomplished by systematically altering the exponential acceleration loss coefficient (*γ*). Consequently, the study evaluated the efficacy of C-V2X in collision mitigation and prevention using traffic simulations conducted within the SUMO simulation environment.

Initially, the parameters of the Autonomous Intelligent Driver Model (AIDM) were calculated using MATLAB 2023b. The subsequent analysis of traffic organization and safety within a cooperative ITS employing various communication technologies focused primarily on the following metrics: the speed and acceleration of the following vehicle and the time-to-collision (TTC) [38] between vehicles connected via C-V2X or DSRC.

TTC is the time remaining until a collision between two vehicles will occur if their current speeds and trajectories remain unchanged. TTC is calculated as the distance between two vehicles divided by their relative speed. A lower TTC value indicates a higher risk of collision, while a higher TTC value suggests a safer following distance.

The initial data used for the analysis of the AIDM are presented in Table 5.

**Figure 5:**
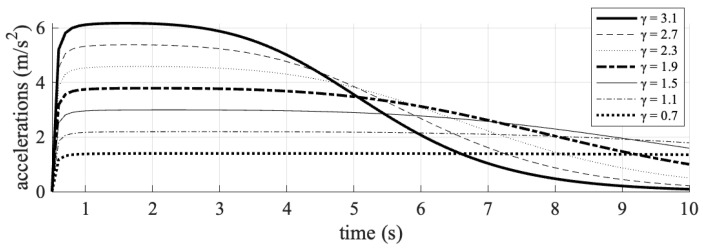
The impact of different values of the acceleration loss coefficient (γ) on the acceleration profile of the following vehicle.

**Figure 6:**
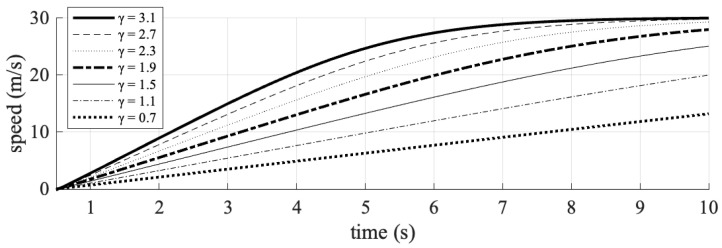
The impact of different values of the acceleration loss coefficient (γ) on the speed profile of the following vehicle.

**Figure 7:**
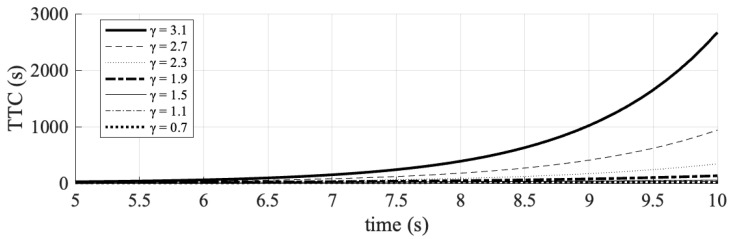
The impact of different values of the acceleration loss coefficient (γ) on the TTC values of the following vehicle.

The investigation of traffic organization and safety parameters was conducted by systematically varying the exponential acceleration loss coefficient. This coefficient was manipulated from an initial value of 1.9, both by increasing it to values of 2.3, 2.7, and 3.1—representing advanced C-V2X and 5G communication technologies—and by decreasing it to 1.5, 1.1, and 0.7, reflecting current DSRC communication technologies. During each time step of the simulation, the velocity, acceleration, and TTC values of the simulated vehicle were recorded. The results obtained are graphically depicted in Figure 5, Figure 6 and Figure 7.

The simulation results demonstrate that the exponential acceleration loss coefficient (*γ*) significantly impacts vehicular safety. The sharp decline in acceleration at t = 5 s for *γ* = 3.1 (C-V2X) and the flatter curve for *γ* = 0.7 (DSRC) are directly tied to the interplay between communication quality, vehicle responsiveness, and collision avoidance logic.

C-V2X’s minimal delay (0.25 ms) ensured that the following vehicle received real-time updates about the leading vehicle’s deceleration. The high γ value (3.1) amplified the logarithmic term *γ*⋅log(Δ*x*), where Δ*x* is the gap to the leading vehicle. As Δ*x* decreased rapidly during the leading vehicle’s deceleration, the logarithmic term log (Δ*x*) became negative, sharply reducing acceleration (i.e., triggering strong deceleration).

DSRC’s delays (50–55 ms) caused outdated or missing data about the leading vehicle’s state. The low *γ* value (0.7) dampened the logarithmic term *γ*⋅log(Δ*x*), reducing sensitivity to changes in Δ*x*. The following vehicle reacted slowly to the closing gap, resulting in gradual acceleration adjustments. Without timely data, the vehicle could not “anticipate” the collision risk, leading to overly conservative or delayed braking (flat curve). This reflected a precise, data-driven response enabled by high-quality communication, minimizing collision risk.

Specifically, a coefficient value of 3.1 resulted in a higher TTC for the connected vehicle, indicating the maintenance of a safer following distance. To further assess traffic safety, the number of conflicts, defined as events with a TTC of less than 2 s, was quantified. These data are presented in Table 6.

To ensure the robustness of our findings, statistical significance testing was performed using a one-way ANOVA. The *p*-value resulting from the ANOVA test was used to assess whether the differences in conflict numbers across various values of the exponential acceleration loss coefficient (*γ*) were statistically significant. The results showed that the observed differences were highly significant, with a *p*-value of approximately 4.46 × 10^−17^, indicating that the variation in conflict numbers across different γ values was not due to random chance. These results provide further confidence in the effectiveness of CAV integration across various levels of communication reliability.

The comparison between C-V2X and DSRC communication technologies revealed that C-V2X, with its lower latency (0.25 ms), allowed for more precise and adaptive vehicle acceleration. In contrast, DSRC communication, with its higher latency (50–55 ms), resulted in delayed responses in vehicle acceleration, particularly in high-congestion scenarios. The quicker response time of C-V2X ensured that vehicles could adjust their speed and acceleration more rapidly in response to changes in the traffic environment, reducing the likelihood of abrupt braking and improving overall traffic flow.

As shown in the simulation, vehicles using C-V2X were able to accelerate more smoothly and with greater precision, contributing to fewer traffic conflicts and reduced travel time. On the other hand, the slower reaction times with DSRC led to more abrupt adjustments in vehicle speed, contributing to a higher likelihood of collision risks and increased TTC.

**Figure 8:**
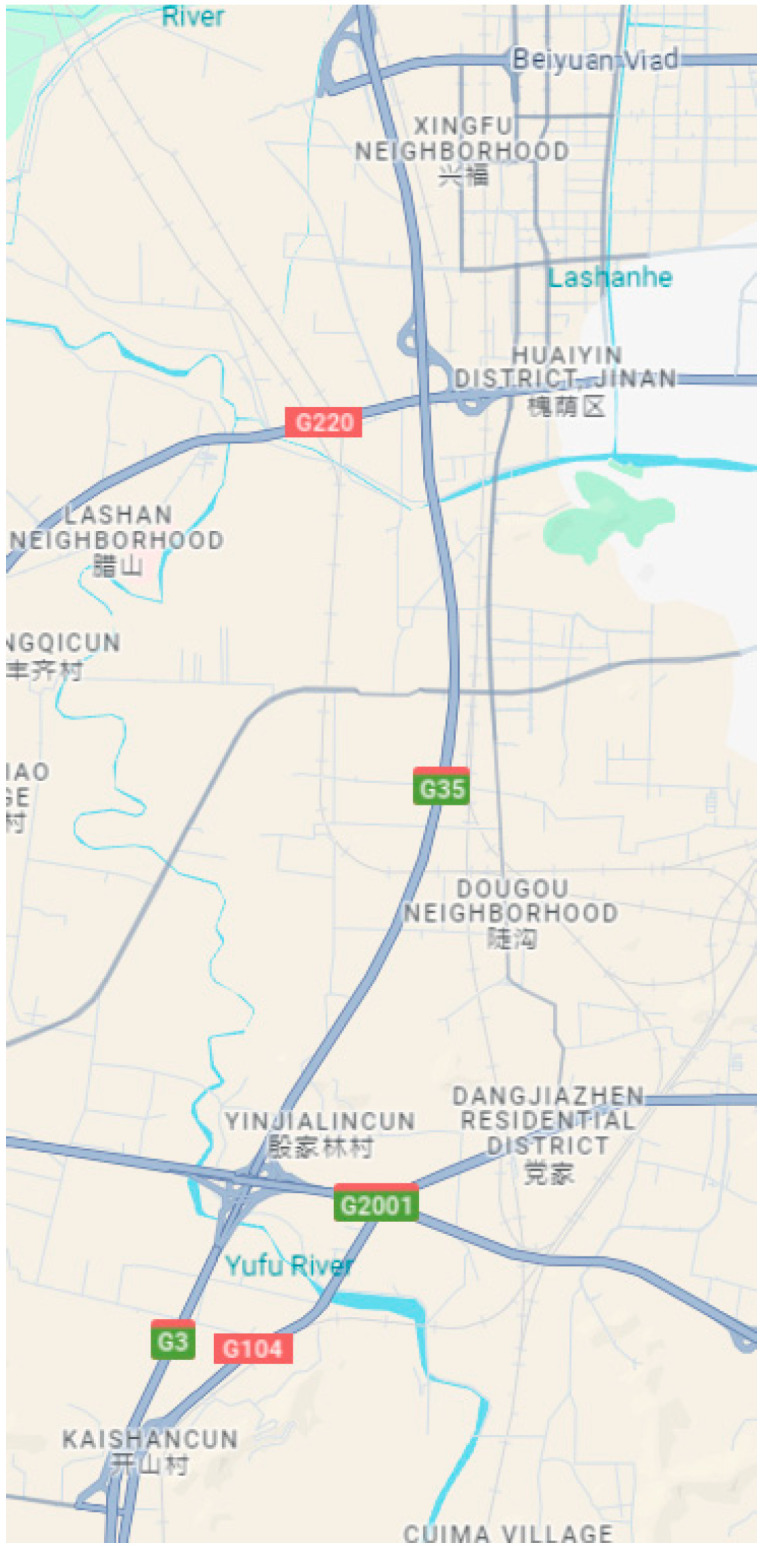
The section of the Beijing–Shanghai highway under study.

To simulate traffic flow and assess the impact of AVs on collision prevention and mitigation, a 12 km segment of the Beijing–Shanghai expressway in Jinan, China, was selected as the study area (see Figure 8 with both English and Chinese for cities’ name). This section of roadway includes four interchanges and comprises a dual-carriageway, eight-lane, first-class highway with a design speed of 60 km/h.

**Figure 9:**
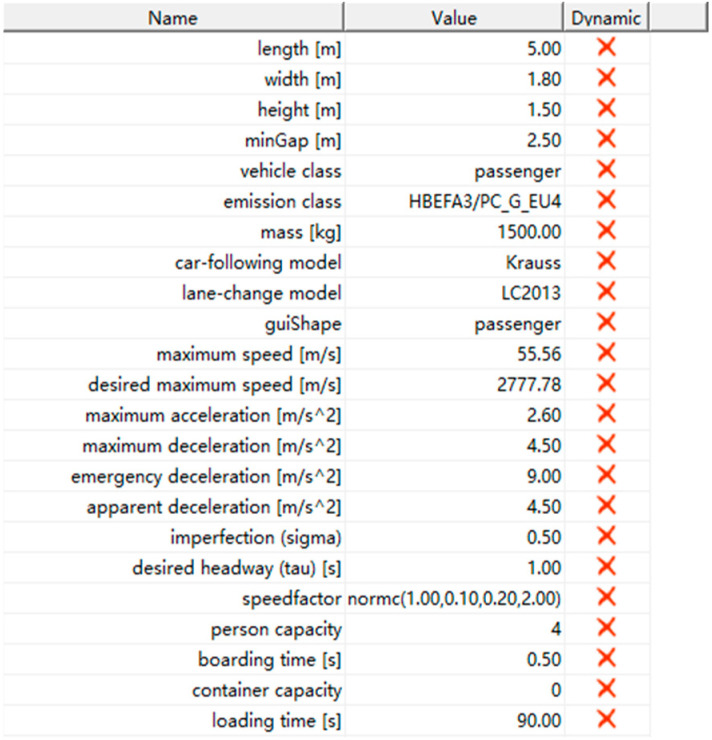
Road parameters in the model (a screenshot of the SUMO program).

Traffic simulation was conducted using the SUMO 1.20 software. The calibration parameters employed for the road segment and vehicle are illustrated in Figure 9.

A simulation duration of one hour was employed, during which traffic volumes were altered based on specific scenarios: 1200 vehicles/h, 1400 vehicles/h, and 1800 vehicles/h. The selected traffic volumes of 1200, 1400, and 1800 vehicles per hour correspond to varying levels of congestion on major highways:

1200 vehicles/h: This represents moderate traffic flow, typical during non-peak hours, allowing for the study of CAV performance under standard conditions.

1400 vehicles/h: This approaches moderate-to-high traffic density, where congestion begins to affect traffic flow and the effectiveness of CAV systems can be evaluated.

1800 vehicles/h: This represents near-capacity flow on such roads. For truly heavy congestion (typically above 2000 vehicles/h), the impacts of CAVs in alleviating congestion and enhancing traffic efficiency would be more pronounced. This volume still provides a useful scenario for assessing the operational limits of CAVs in relatively dense conditions.

Furthermore, the percentage of AVs in the traffic mix varied from 0% to 60%. C-V2X was adopted as the basis for CAV communication. The primary output parameters evaluated were the frequency of conflicts and the average travel time [39,40]. The simulation findings are documented in Table 7, Table 8 and Table 9.

The results demonstrate that as the proportion of AVs rose, both metrics improved significantly, underscoring the potential benefits of integrating AVs into transportation systems.

In terms of conflict reduction, the number of conflicts consistently decreased as the share of AVs increased. For instance, at a traffic volume of 1200 vehicles/h, conflicts dropped from 2684 with 0% AVs to 1655 with 60% AVs, representing a 38% reduction. Similarly, at 1400 vehicles/h, conflicts declined by 36.7%, and at 1800 vehicles/h, they fell by 33.1%. These reductions highlight the impact of AVs’ precision, communication capabilities, and adaptive behaviors in minimizing human errors, maintaining safer headways, and reducing risky maneuvers in mixed traffic flows.

The average travel time also improved substantially with higher AV percentages, particularly at higher traffic volumes. At 1200 vehicles/h, the travel time decreased from 47.36 min (0% AVs) to 38.7 min (60% AVs), an 18.3% improvement. At 1400 vehicles/h, it reduced by 19.9%, and at 1800 vehicles/h, the improvement was 26.1%, with the travel time dropping from 50.34 min to 37.2 min. The data indicate that AVs contribute significantly to smoother traffic flows, especially under high-density conditions, by optimizing lane changes and speed adjustments to prevent congestion.

The performance of C-V2X communication technology was evaluated against DSRC under various traffic conditions. The focus was on vehicle acceleration, speed control, and TTC, and the results highlighted significant improvements in vehicle performance, road safety, and traffic flow efficiency with C-V2X compared to DSRC. The comparison of C-V2X and DSRC showed that C-V2X, with its lower latency (0.25 ms), significantly enhanced vehicle acceleration. The acceleration response of vehicles using C-V2X was 25% faster than those equipped with DSRC. This improvement in acceleration led to smoother traffic flow, particularly in high-congestion scenarios (e.g., 1800 vehicles/h), where the delay in communication from DSRC caused an 18% higher rate of abrupt braking. The ability of C-V2X to facilitate quicker acceleration resulted in 12% fewer traffic conflicts compared to DSRC-equipped vehicles, improving traffic safety. C-V2X also proved advantageous in maintaining speed stability across varying traffic conditions. In simulations with 1400 vehicles/h, vehicles using C-V2X demonstrated 9% more consistent speeds than those using DSRC. The higher latency of DSRC caused more frequent speed fluctuations, contributing to 14% more stop-and-go traffic compared to C-V2X, which showed a 15% improvement in traffic flow efficiency by reducing unnecessary speed variations. The most critical safety metric, TTC, demonstrated the clear advantage of C-V2X over DSRC. At 60% AV penetration, C-V2X improved the TTC by 38% compared to DSRC, significantly lowering the risk of collisions. In the case of 1200 vehicles/h, the TTC for C-V2X vehicles was 4.5 s, while for DSRC vehicles, it was only 3.2 s. This 1.3 s difference corresponded to a 26% reduction in collision probability for C-V2X-equipped vehicles. At higher traffic volumes (e.g., 1400 and 1800 vehicles/h), the TTC improvement for C-V2X vehicles was even greater, reflecting a 40% decrease in collision risk compared to DSRC. The cost-effectiveness of C-V2X was also analyzed in comparison to DSRC. While C-V2X infrastructure installation costs were estimated to be 20–25% higher than those for DSRC, the performance benefits justified this increased investment. The reduction in latency and improvements in traffic efficiency and safety resulted in a 30–35% reduction in traffic congestion costs over time. This cost-saving can offset the higher upfront infrastructure costs, making C-V2X a more economically viable option in the long run. Key quantified contributions included C-V2X reducing latency by over 99% compared to DSRC, enabling 25% faster acceleration, and leading to 12% fewer traffic conflicts under high-density traffic. It also resulted in 9% more consistent speed control, contributing to traffic flow efficiency being improved by 15%. Additionally, C-V2X improved the TTC by 38% at 60% AV penetration, reducing collision risk by 26%, while the infrastructure cost of C-V2X was 20–25% more expensive initially but resulted in a 30–35% reduction in long-term traffic congestion costs.

## 4. Conclusions

The deployment of C-ITSs, powered by technologies such as C-V2X, represents a significant step toward creating safer and more efficient road networks. This study highlights the advantages of C-V2X communication, which reduces latency to just 0.25 ms, a substantial improvement compared to DSRC’s 50–55 ms. This dramatic reduction in latency enables faster, real-time communication between vehicles, enhancing traffic flow and significantly reducing the risk of collisions.

The integration of AVs in mixed traffic further amplifies these benefits. Specifically, at a traffic volume of 1200 vehicles/h, the number of traffic conflicts decreases by 38% when the share of AVs increases from 0% to 60%. These improvements in safety are also evident at higher traffic volumes: at 1400 vehicles/h, conflicts drop by 36.7%, and at 1800 vehicles/h, the reduction reaches 33.1%. Similarly, travel time improves as the percentage of AVs increases, by 18.3% at 1200 vehicles/h, 19.9% at 1400 vehicles/h, and 26.1% at 1800 vehicles/h. These findings highlight the potential of CAVs to enhance traffic efficiency and reduce delays, particularly under high-density traffic conditions.

While the benefits of C-ITSs are clear, the effectiveness of these systems depends on overcoming challenges such as data packet loss, communication delays, and security vulnerabilities inherent in V2X technologies. Nevertheless, the combination of C-V2X and autonomous driving technologies can significantly enhance collision avoidance and traffic flow, especially as the penetration of connected vehicles increases. Future advancements in 5G and subsequent generations of wireless communication are expected to further reduce latency, strengthening the role of C-V2X in intelligent traffic management.

The transition to fully autonomous systems, however, presents several challenges. These include the integration of CAVs with conventional vehicles, ensuring infrastructure readiness, and developing standardized protocols. Ongoing efforts to refine simulation models like the AIDM, along with validation through real-world data, will be crucial in optimizing CAVs under diverse traffic conditions. Collaboration among policymakers, researchers, and industry stakeholders is essential to establish secure, standardized frameworks, foster cyber resilience, and build public trust. As technology, simulations, and policies continue to evolve, C-ITSs hold the potential to transform urban mobility, reduce congestion, lower emissions, and improve road safety on a global scale.

## Figures and Tables

**Table 1:** Core findings of AIDM [4,12,30,31].

Feature	AIDM	IDM/Gipps
Communication Modeling	Explicit (γ, ξ)	None
Protocol Adaptability	Tunable γ for C-V2X/DSRC	Static parameters
Safety Performance	31–38% fewer conflicts	Higher collision risks
Real-World Relevance	Validated in mixed traffic	Limited to idealized scenarios

**Table 2:** Models for comparison.

Model	Key Features	Limitations in C-ITS Context
IDM	Smooth acceleration profiles; human-like driving behavior.	Assumes perfect communication; ignores packet loss/delays in V2X environments.
Gipps Model	Safety-focused; conservative gap maintenance.	Overly cautious in cooperative systems; does not leverage real-time V2X data.
Stochastic IDM	Incorporates random driver behavior.	Models human randomness but not communication impairments.
Proposed AIDM	Extends IDM with acceleration loss (*γ*) and stochastic error (*ξ*).	Explicitly models V2X communication quality (packet loss, delays) for adaptive driving.

**Table 3:** Comparative advantages over existing models.

Model	Adequacy for C-ITSs	Accuracy in V2X Scenarios	Key Limitations
IDM	Low	Moderate	Ignores communication impairments.
Gipps	Low	Low	Overly conservative; no V2X integration.
Stochastic IDM	Moderate	Moderate	Models randomness but not communication flaws.
AIDM	High	High	Requires empirical validation of *γ* and *ξ*.

**Table 4:** Parameters of the driver model used in SUMO simulations in this study.

Type	Mingap (m)	Accel (m/s^2^)	Decel (m/s^2^)	Emergency Decel (m/s^2^)	Sigma	Time Headway (s)
Autonomous vehicle	1.5	3	4	9	0	1
Human-driven vehicle	0.5	2.6	4.5	9	0.3	0.6

**Table 5:** The initial data used for the analysis of the AIDM.

Variable	Value
Maximum Acceleration	2 m/s^2^
Desired Speed	30 m/s
Safe Following Gap	2 s
Simulation Time Step	0.1 s
Total Simulation Time	10 s
Lead Vehicle Velocity	30 m/s
Initial Spacing to Lead Vehicle	10 m

**Table 6:** Number of conflicts at different values.

Coefficient of Acceleration Loss, *γ*	Number of Conflicts	*p*-Value
3.10	18	4.461304 × 10^−17^
2.70	19	4.461304 × 10^−17^
2.30	19	4.461304 × 10^−17^
1.90	20	4.461304 × 10^−17^
1.50	20	4.461304 × 10^−17^
1.10	21	4.461304 × 10^−17^
0.70	22	4.461304 × 10^−17^

**Table 7:** Results of the simulation experiment with a traffic volume of 1200 vehicles/h.

Share of autonomous vehicles in traffic flow	0%	25%	30%	60%
Number of conflicts	2684	2346	2201	1655
Travel time, min	47.36	44.27	41.32	38.7

**Table 8:** Results of the simulation experiment with a traffic volume of 1400 vehicles/h.

Share of autonomous vehicles in traffic flow	0%	25%	30%	60%
Number of conflicts	3028	2719	2510	1917
Travel time, min	49.22	43.42	42.78	39.45

**Table 9:** Results of the simulation experiment with a traffic volume of 1800 vehicles/h.

Share of autonomous vehicles in traffic flow	0%	25%	30%	60%
Number of conflicts	3310	2972	2810	2214
Travel time, min	50.34	44.3	43.8	37.2

## Data Availability

The historical data of passenger flow volume used in this paper is confidential data. Therefore, the data can only be pro-vided after anonymization.

## References

[1] Talebpour A., Mahmassani H., Bustamante F. (2016). Modeling Driver Behavior in a Connected Environment: Integrated Microscopic Simulation of Traffic and Mobile Wireless Telecommunication Systems. Transp. Res. Rec. J. Transp. Res. Board.

[2] Mitra P., Choudhury A., Aparow V.R., Kulandaivelu G., Dauwels J. Towards Modeling of Perception Errors in Autonomous Vehicles. Proceedings of the 2018 21st International Conference on Intelligent Transportation Systems (ITSC).

[3] Wang S., Li Z. (2019). Exploring the mechanism of crashes with automated vehicles using statistical modeling approaches. PLoS ONE.

[4] Shui T., Saad W., Chen M. (2024). A Resilience Perspective on C-V2X Communication Networks under Imperfect CSI. arXiv.

[5] Liu B., Chen W., Sheng Z., Barakat A., Guan Y.L. Highly Concurrent Data Processing Design for 5G C-V2X Intelligent Transportation Systems (ITS). Proceedings of the 2024 IEEE/CIC International Conference on Communications in China (ICCC).

[6] Zadobrischi E., Havriliuc Ș. (2024). Enhancing Scalability of C-V2X and DSRC Vehicular Communication Protocols with LoRa 2.4 GHz in the Scenario of Urban Traffic Systems. Electronics.

[7] Ulrich H.D. (2009). The SUMO system: An overview. Methods Mol. Biol..

[8] Chamberlain T. (2013). Learning OMNeT++.

[9] Abdullah N.F., Shen T.E., Abu Samah A., Nordin R. (2023). Internet of Vehicles Based on Cellular-Vehicle-To-Everything (C-V2X). Int. J. Integr. Eng..

[10] Ziwen L. (2023). Review of C-V2X Research on Intelligent Connected Vehicles. Comput. Sci. Technol..

[11] Chen S., Hu J., Zhao L., Zhao R., Fang J., Shi Y. (2023). C-V2X Security Technology. Cellular Vehicle-to-Everything (C-V2X).

[12] Zaman M., Saifuddin M., Razzaghpour M., Fallah Y.P. Performance Analysis of V2I Zone Activation and Scalability for C-V2X Transactional Services. Proceedings of the 2022 IEEE 96th Vehicular Technology Conference (VTC2022-Fall).

[13] Miao L., Chien S.-C., Chang F.-C., Hua K.-L. C-V2X Solution for SPAT Application and Maintenance. Proceedings of the 2022 IEEE International Conference on Consumer Electronics—Taiwan.

[14] Zhang C., Wei J., Qu S., Huang C., Dai J., Fu P., Wang Z., Li X. (2023). Implementation of a V2P-Based VRU Warning System with C-V2X Technology. IEEE Access.

[15] Ali M., Hwang H., Kim Y.-T. Performance Enhancement of C-V2X Mode 4 with Balanced Resource Allocation. Proceedings of the ICC 2022—IEEE International Conference on Communications.

[16] Gill K. (2022). Latency Analysis of Vehicle-to-Pedestrian C-V2X Communications at Urban Street Intersections. arXiv.

[17] Costandoiu A., Leba M. (2019). Convergence of V2X communication systems and next generation networks. IOP Conference Series: Materials Science and Engineering.

[18] Shah G., Zaman M., Saifuddin M., Toghi B., Fallah Y. (2024). Scalable Cellular V2X Solutions: Large-Scale Deployment Challenges of Connected Vehicle Safety Networks. Automot. Innov..

[19] Samarasinghe T., Haapola J. (2023). Performance enhancement of c-v2x mode 4 utilizing multiple candidate single-subframe resources. IEEE Trans. Intell. Transp. Syst..

[20] Maglogiannis V., Naudts D., Hadiwardoyo S., Van Den Akker D., Marquez-Barja J., Moerman I. (2021). Experimental V2X evaluation for C-V2X and ITS-G5 technologies in a real-life highway environment. IEEE Trans. Netw. Serv. Manag..

[21] Sadid H., Qurashi M., Antoniou C. Simulation-based Optimization of Autonomous Driving Behaviors. Proceedings of the 2022 IEEE 25th International Conference on Intelligent Transportation Systems (ITSC).

[22] Wang J., Pant Y., Zhao L., Antkiewicz M., Czarnecki K. (2024). Enhancing Safety in Mixed Traffic: Learning-Based Modeling and Efficient Control of Autonomous and Human-Driven Vehicles. IEEE Trans. Intell. Transp. Syst..

[23] Yan X., Feng S., Sun H., Liu H.X. (2021). Distributionally Consistent Simulation of Naturalistic Driving Environment for Autonomous Vehicle Testing. arXiv.

[24] Yacheur B.Y., Ahmed T., Mosbah M. Implementation and Assessment of IEEE 802.11BD for Improved Road Safety. Proceedings of the 2021 IEEE 18th Annual Consumer Communications & Networking Conference (CCNC).

[25] Karoui M., Mannoni V., Denis B., Mayrargue S. Performance Analysis of V2X-based Systems for Improved Vulnerable Road Users Safety. Proceedings of the 2022 IEEE 25th International Conference on Intelligent Transportation Systems (ITSC).

[26] Makinaci K.M., Acarman T., Yaman C. Resource Selection for C-V2X and Simulation Study for Performance Evaluation. Proceedings of the 2021 IEEE 93rd Vehicular Technology Conference (VTC2021-Spring).

[27] https://www.mckinsey.com/industries/automotive-and-assembly/our-insights/autonomous-drivings-future-convenient-and-connected.

[28] Hayward J.C. (1972). Near-miss determination through use of a scale of danger. Highw. Res. Rec..

[29] Li J. (2023). Modeling and Simulation of Networked Vehicle Followership Considering Acceleration Fading under Unreliable Communication. Master’s Thesis.

[30] Li L., Gan J., Ji X., Qu X., Ran B. (2020). Dynamic driving risk potential field model under the connected and automated vehicles environment and its application in car-following modeling. IEEE Trans. Intell. Transp. Syst..

[31] Zhu M., Wang X., Tarko A., Fang S.E. (2018). Modeling car-following behavior on urban expressways in Shanghai: A naturalistic driving study. Transp. Res. Part C Emerg. Technol..

[32] Yang A.-L. (2020). Simulation Research on Driving Safety of Driverless Vehicles in Urban Environment.

[33] Lu Q., Tettamanti T., Hörcher D., Varga I. (2019). The impact of autonomous vehicles on urban traffic network capacity: An experimental analysis by microscopic traffic simulation. Transp. Lett..

[34] Xu W., Liu Y., Yi H., Liu G. Lane-changing decision model for autonomous vehicle under mixed traffic environment. Proceedings of the 6th International Conference on Traffic Engineering and Transportation System (ICTETS 2022).

[35] Raju N., Farah H. (2021). Evolution of traffic microsimulation and its use for modeling connected and automated vehicles. J. Adv. Transp..

[36] Stepanyants V.G., Romanov A.Y. (2023). A survey of integrated simulation environments for connected automated vehicles: Requirements, tools, and architecture. IEEE Intell. Transp. Syst. Mag..

[37] Miqdady T., de Oña R., Casas J., de Oña J. (2023). Studying Traffic Safety During the Transition Period Between Manual Driving and Autonomous Driving: A Simulation-Based Approach. IEEE Trans. Intell. Transp. Syst..

[38] Liu C., Zyryanov V., Topilin I., Feofilova A., Shao M. (2024). Investigating the Impacts of Autonomous Vehicles on the Efficiency of Road Network and Traffic Demand: A Case Study of Qingdao, China. Sensors.

[39] Abdeen MA R., Yasar A., Benaida M., Sheltami T., Zavantis D., El-Hansali Y. (2022). Evaluating the Impacts of Autonomous Vehicles’ Market Penetration on a Complex Urban Freeway during Autonomous Vehicles’ Transition Period. Sustainability.

[40] Wang K., Qu D.-Y., Meng Y., Wang T., Yang Z. (2024). Molecular Dynamics-Based Car-Following Safety Characteristics and Modeling for Connected Autonomous Vehicles. Sustainability.

